# Evaluation of periodontally diseased molars based on the Miller McEntire Periodontal Prognostic Index

**DOI:** 10.1080/07853890.2021.1897342

**Published:** 2021-09-28

**Authors:** Vanessa Machado, João Botelho, Luís Proença, Ricardo Alves, Maria Alzira Cavacas, Luís Amaro, José João Mendes

**Affiliations:** a Periodontology Department, Clinical Research Unit (CRU), Centro de Investigação Interdisciplinar Egas Moniz (CiEM), Egas Moniz-Cooperativa de Ensino Superior, Caparica, Portugal; b Clinical Research Unit (CRU), Centro de Investigação Interdisciplinar Egas Moniz (CiiEM), Egas Moniz Cooperativa de Ensino Superior, Caparica, Portugal; c Quantitative Methods for Health Research (MQIS), Centro de Investigação Interdisciplinar Egas Moniz (CiiEM), Egas Moniz Cooperativa de Ensino Superior, Caparica, Portugal; d Health Centers grouping (HCG) Almada-Seixal, Regional Health Administration of Lisbon and Tagus Valley (RHALTV), Portugal; e Hospital Garcia de Orta, Almada, Portugal

## Abstract

**Introduction:**

The Miller McEntire Periodontal Prognostic Index (MMPPI) is a simple, powerful, evidenced-based, statistically validated, and accurate motivational tool that provides the periodontal prognosis on molar teeth [1]. This study aimed to evaluate and establish the periodontal prognosis and risk assessment of diseased molars, based on the MMPPI, in a sample of patients from a population-based epidemiologic survey carried out in the southern Lisbon Metropolitan Area.

**Materials and methods:**

From December 2018 to April 2019, data were collected on 4,063 molars from a total of 1,064 patients, by two calibrated examiners (J.B. and V.M.). The considered prognosis parameters for MMPI calculation were: age, furcation involvement, smoking, probing depth (mm), mobility and molar type [2]. An MMPPI score higher than six was considered representative of a molar tooth at high risk. Potential risk factors, such as gender, dental arch, and quadrant location were assessed by logistic regression modelling. Univariate and adjusted multivariate odds ratio (OR) and correspondent 95% confidence intervals (95% CI) were determined. This study was approved by the ARSLVT Ethics Committee (3525 & 8696/CES/2018).

**Results:**

From the 4,063 molars present at the time of observation (47.7% from total), 202 (5.0%, 95% CI: 4.3–5.7%) were identified as being at high risk. Overall, the MMPPI score ranged from 0 to 12 with a median of 2. A logistic regression model was fitted to the data. Within the model, being located on the upper arch (OR = 8.9, 95% CI: 5.4–14.7) and belonging to a male patient OR = 2.0, 95% CI: 1.5–2.6), were the factors significantly associated with a tooth at high risk.

**Discussion and conclusions:**

This index contributes to a more informed prognostic assessment of periodontally compromised molars [1]. Male patients and upper molars are the main risk factors that increase the likelihood of developing periodontal problems in molars. These results highlight potential targeting for public health measures.
